# Anti‐PF4 mediated thrombocytopenia and thrombosis associated with acute cytomegalovirus infection displays both HIT‐like and VITT‐like characteristics

**DOI:** 10.1111/bjh.20092

**Published:** 2025-04-29

**Authors:** Phillip L. R. Nicolson, Samantha J. Montague, Richard J. Buka, Anthony Calvert, Jo‐Ann I. Sheppard, Yi Zhang, Jing Jing Wang, Jack Sharman, Eman Hassan, James Harrison, Errin Lawrence, Phillip El‐Dalil, Dhruv Parekh, Husam Osman, Tom P. Gordon, Ishac Nazy, Theodore E. Warkentin, Will A. Lester

**Affiliations:** ^1^ Department of Haemostasis, Liaison and Transfusion Haematology Queen Elizabeth Hospital Birmingham UK; ^2^ Department of Cardiovascular Sciences, College of Medicine and Health University of Birmingham Birmingham UK; ^3^ H&I, NHS Blood and Transplant Bristol UK; ^4^ Department of Pathology and Molecular Medicine Michael G. DeGroote School of Medicine, McMaster University Hamilton Ontario Canada; ^5^ McMaster Center for Transfusion Research, McMaster University Hamilton Ontario Canada; ^6^ Department of Immunology, College of Medicine and Public Health Flinders University Bedford Park South Australia Australia; ^7^ Department of Immunology SA Pathology, Flinders Medical Centre Bedford Park South Australia Australia; ^8^ Department of Critical Care Queen Elizabeth Hospital Birmingham UK; ^9^ Department of Inflammation and Ageing, College of Medicine and Health University of Birmingham Birmingham UK; ^10^ Department of Virology Birmingham Heartlands Hospital Birmingham UK; ^11^ Department of Biochemistry and Biomedical Sciences McMaster University Hamilton Ontario Canada; ^12^ Department of Medicine Michael G. DeGroote School of Medicine, McMaster University Hamilton Ontario Canada; ^13^ Department of Medicine Hamilton Health Sciences Hamilton Ontario Canada

**Keywords:** cytomegalovirus, FCY receptor, heparin‐induced TP, monoclonal antibodies, platelet activation, platelet factor 4, thrombocytopenia, thrombosis

## Abstract

Vaccine‐induced immune thrombocytopenia and thrombosis (VITT) is one of several anti‐platelet factor 4 (anti‐PF4)‐associated immune thrombocytopenia and thrombosis (PITT) syndromes. As well as following adenoviral vector vaccines, VITT has recently been described following acute adenovirus infection. We describe a patient with PITT following acute cytomegalovirus infection. The antibody clonotype and PF4 epitopes were distinct from those identified in VITT, and they were detectable as a paraprotein. PITT should be considered in all patients with thrombocytopenia and thrombosis, even without preceding vaccination or heparin, but who otherwise meet the VITT criteria defined by the British Society of Haematology Expert Panel.

## INTRODUCTION

Vaccine‐induced immune thrombocytopenia and thrombosis (VITT) was first described in early 2021 soon after the roll out of adenoviral vector COVID‐19 vaccines (Oxford‐AstraZeneca ChAdOx1 nCoV‐19 [AZD1222] and Janssen/Johnson & Johnson [Ad26.COV2.S]).^1^, ^2^, ^3^ Patients presented with thrombosis, often at unusual sites, thrombocytopenia and features of disseminated intravascular coagulation (DIC) with low fibrinogen and high D‐dimer levels 5–30 days following vaccination.^4^

The condition is characterized by high levels of anti‐platelet factor 4 (PF4) autoantibodies detectable by enzyme‐linked immunosorbent assay (ELISA) but not by rapid heparin‐induced thrombocytopenia (HIT) assays such as the ACL AcuStar chemiluminescence assay (Werfen, Spain).^1^, ^5^ Functional platelet testing by activation of healthy donor platelets by VITT serum in the serotonin release assay (PF4‐SRA) or the PF4‐induced platelet activation (PIPA) test is typically blocked by low concentrations (~0.5 U/mL) of heparin but enhanced by PF4.^6^, ^7^ This contrasts with HIT and autoimmune HIT, where chemiluminescence assays for anti‐PF4/heparin antibodies are positive and platelet activation is enhanced by low concentrations of heparin in SRA, the heparin‐induced platelet activation (HIPA) and commercial HITAlert™ tests.^7^, ^8^

Elegant studies have shown that the anti‐PF4 antibodies in HIT and VITT bind differently to PF4. In HIT, heparin binding induces a conformational change in PF4 permitting antibody binding, while in VITT, antibodies bind to the native protein without a conformational change.^9^, ^10^

VITT is treated with non‐heparin anticoagulation, intravenous immunoglobulin (IVIg) and/or plasma exchange (PEX) in particularly resistant cases.^11^ There has been speculation that heparin‐based anticoagulation may be beneficial in VITT because of its blocking effect on in vitro functional platelet assays.^12^

It is known that a HIT‐like syndrome can occur without heparin exposure. Recently it has become clear that a VITT‐like syndrome can occur without vaccine exposure.^8^, ^13^ Warkentin et al. published two cases of adenovirus‐associated thrombocytopenia and thrombosis with VITT‐like antibodies that bound to the same region of PF4 and had identical amino acid sequences as those seen in VITT.^13^, ^14^ Other groups have also published similar cases, some with proximate adenoviral infection.^15^, ^16^, ^17^, ^18^

We report a patient who presented with portal vein thrombosis and thrombocytopenia that worsened following heparin exposure. She was found to have anti‐PF4 antibodies that tested as both VITT‐like and HIT‐like in different assays. Further studies showed this was a novel monoclonal antibody that bound to an overlap region between VITT and HIT antibody binding sites as well as a novel epitope on PF4 not previously described. Further unique features include no proximate adenoviral vaccine or adenovirus exposure, recent cytomegalovirus (CMV) infection and high‐titre anti‐PF4 antibody, detectable as a paraprotein.

## METHODS

Written consent was obtained from next of kin in line with the Declaration of Helsinki. Blood collection was authorized under research ethics approvals 15/NW/0079 and University of Birmingham Internal Ethical Review (ERN_11‐0175). Methodology for in vitro functional platelet experiments and patient serum isolation are detailed in Supporting Information.

## CASE PRESENTATION

For a full case history, see Supporting Information. Briefly, a 37‐year‐old female with no significant medical history presented with fevers and abdominal pain. Her blood tests showed thrombocytopenia, a low fibrinogen and a very high D‐dimer, as well as a mildly raised alanine transaminase and C‐reactive protein. A full panel of investigation results are shown in Table S1. Imaging revealed a portal vein thrombosis. Prophylactic dose low molecular weight heparin (LMWH) was started. Screens for antiphospholipid syndrome, paroxysmal nocturnal haemoglobinuria (PNH) and myeloproliferative disease were negative. There was no bleeding following prophylactic LMWH, so treatment was escalated to a therapeutic dose. Soon afterwards, she infarcted her right middle cerebral artery, liver and spleen. The portal vein thrombosis extended resulting in small bowel ischaemia. She had emergency small bowel resection and was switched to unfractionated heparin (UFH). A CT venogram ruled out cerebral venous sinus thrombosis (CVST).

Despite no recent history of vaccination or heparin treatment, the aggressive thrombosis and thrombocytopenia triggered consideration of PF4‐associated immune thrombocytopenia and thrombosis; Chemiluminescence testing for anti‐PF4 antibodies was negative, but ELISA testing was strongly positive (>3.0 OD) with 98% neutralization with 0.5 U/mL heparin. She was treated with intravenous corticosteroids, but before she could be switched to non‐heparin anticoagulation and undergo plasma exchange, she suffered a cardiac arrest. Resuscitation was unsuccessful. She sadly died only 4 days after presentation.

**FIGURE 1:**
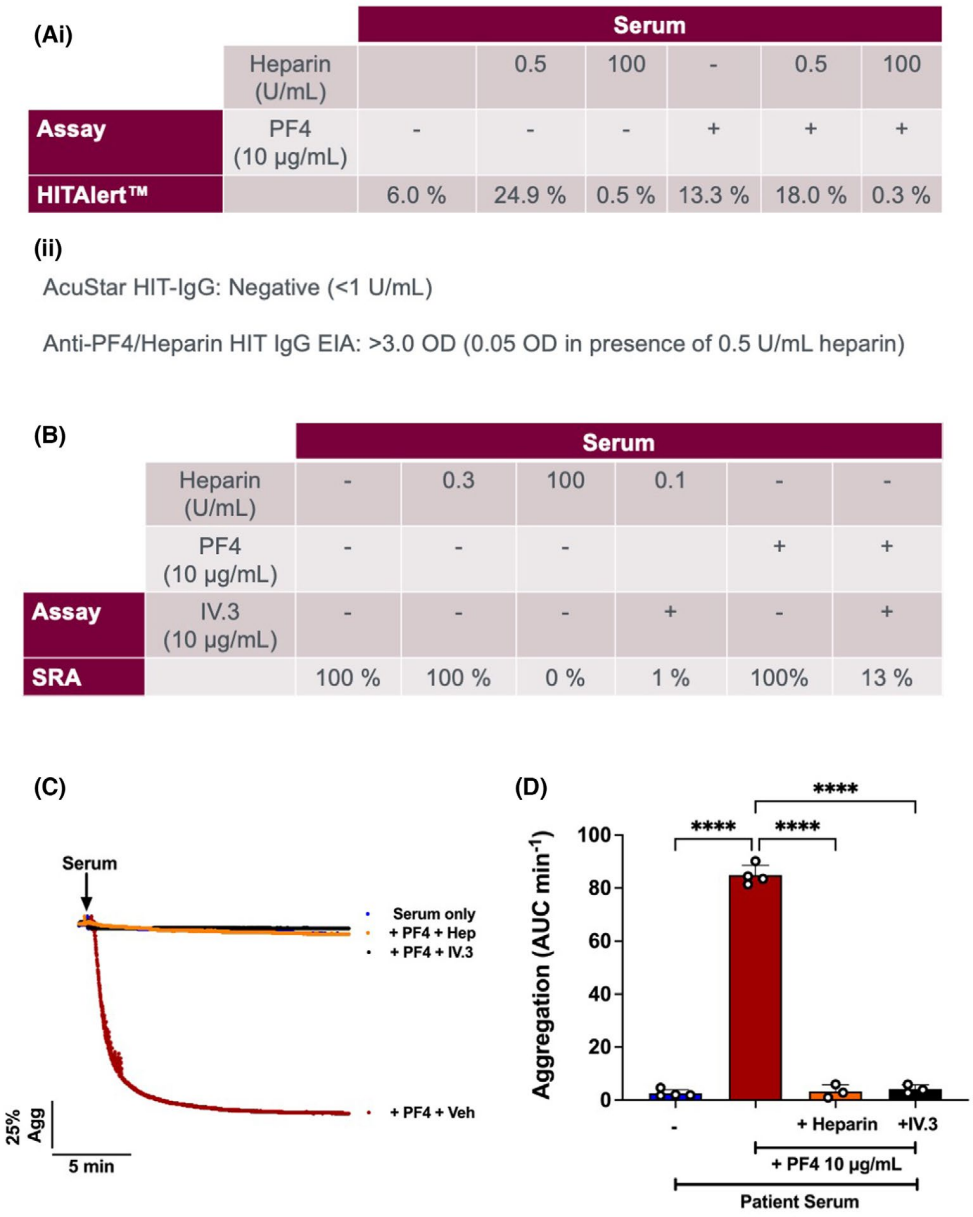
Platelet function testing shows heparin‐induced thrombocytopenia (HIT)‐like, vaccine‐induced immune thrombocytopenia and thrombosis (VITT)‐like or a mixed picture depending on testing modality. Healthy donor platelet‐rich plasma (PRP) was stimulated with patient serum with/without heparin and/or platelet factor 4 (PF4) (A) in the presence of a fluorescein isothiocyanate (FITC)‐conjugated activation marker antibody (as denoted by the HITAlert™ Kit package insert). Percentage of platelets positive for P‐selectin was measured by flow cytometry (Ai). Chemiluminescence AcuStar™ HIT‐IgG and LIFECODES™ Anti‐PF4/Heparin HIT IgG enzymatic immunoassay (EIA) (Aii).^14^ C‐Serotonin loaded healthy donor washed platelets (3 ×10^8^/mL) were stimulated with patient serum with/without heparin and/or PF4 and/or IV.3. The supernatant was then analysed for radioactivity using a β‐counter (B). Healthy donor washed platelets (2 × 10^8^/mL) were stimulated with serum ± PF4 (10 μg/mL) ± Heparin (0.5 U/mL), IV.3 (10 μg/mL) or vehicle (PBS). Representative aggregation trace (C) and mean data (D) shown. Data presented as maximum aggregation (area under the curve; AUC/min). Mean + SD, *n* = 3–5, statistical analysis by one‐way analysis of variance (ANOVA). ****p < 0.0001.

**FIGURE 2:**
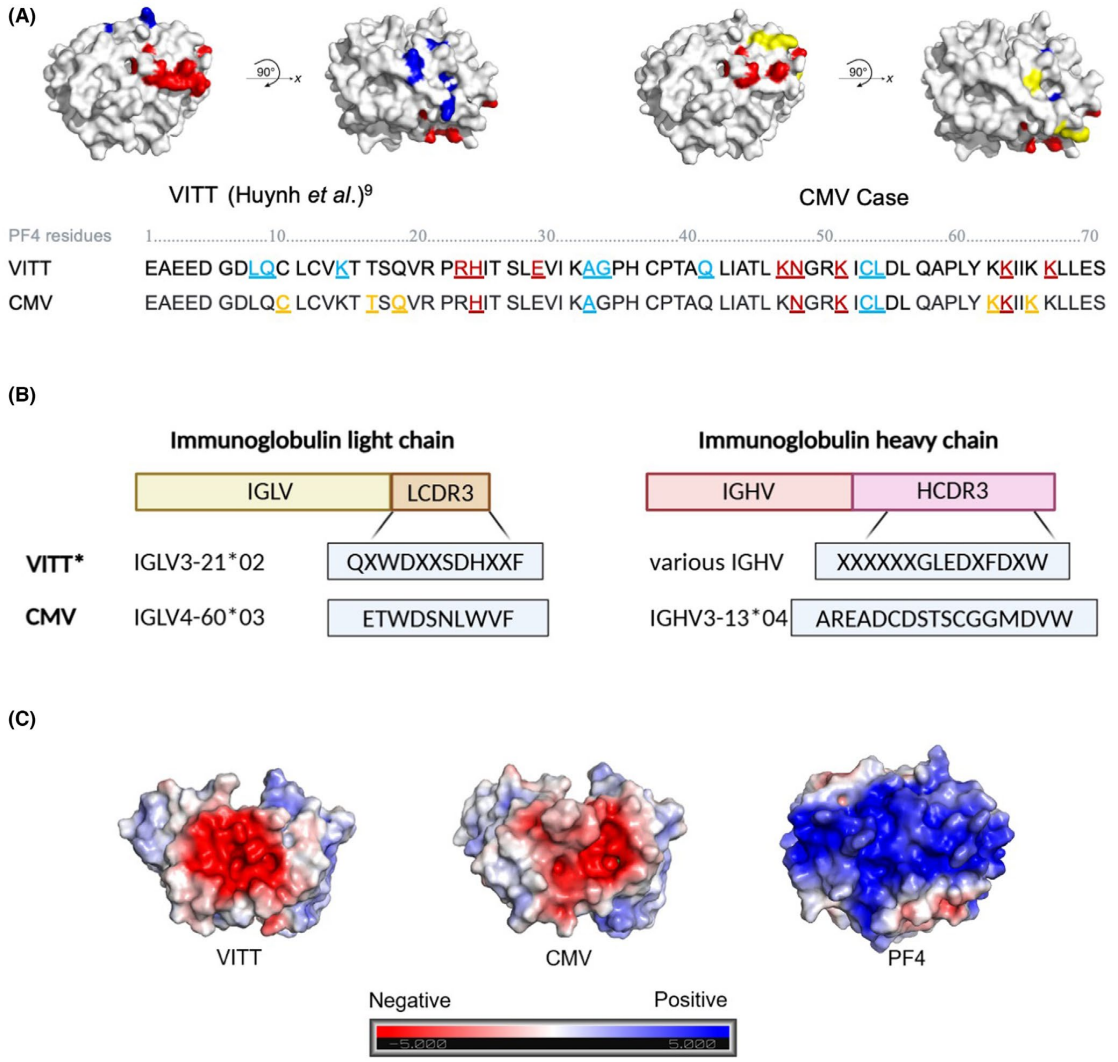
Anti‐platelet factor 4 (anti‐PF4) epitope mapping and proteomic analysis show distinct PF4 binding sites and anti‐PF4 clonotypes between patients with vaccine‐induced immune thrombocytopenia and thrombosis (VITT) and this patient following cytomegalovirus infection. Antibodies show similar negatively charged antigen‐binding regions to those with VITT. Serum was added to ELISA plates coated with wild‐type or mutant PF4, and the degree of anti‐PF4 binding was assessed using an alkaline phosphatase‐conjugated secondary antibody as previously described^9^ (A). Residues previously identified as binding sites for PF4‐dependent VITT antibodies are shown in red, and those identified as binding sites for PF4‐independent VITT antibodies are shown in blue.^10^ Residues distinct from previously recognized sites are shown in yellow. Patients with VITT show a stereotypic clonotype featured by an identical immunoglobulin lambda variable 3‐21*02 (IGLV3‐21*02) light chain paired with a single heavy chain expressing a shared clonotypic ‘GLED’ amino acid motif.^20^ In comparison, this patient shows different immunoglobulin light and heavy chain molecular signatures. (IGLV, immunoglobulin lambda variable; LCDR3, light‐chain complementarity‐determining region 3; IGHV, immunoglobulin heavy variable; HCDR3, heavy‐chain complementarity‐determining region) (B). Paratope modelling for VITT and this patient's anti‐PF4 antibodies show a similar negatively charged antigen‐binding regions that electrostatically match the positively charged PF4 tetramer (C). The scale bar indicates surface electrostatic potential and corresponds to acidic (red) and basic (blue) amino acid residues. *indicates data previously shown in Wang et al. Blood 2022 and Wang et al. New Eng J Med 2024.^14^, ^19^

Virology results available post‐mortem were negative for COVID‐19, Respiratory Syncytial Virus, Influenza A and B, HIV, Hepatitis B and C Virus. Epstein–Barr virus nuclear antigen (EBNA) antibodies were positive, indicating past EBV infection. CMV IgM and low avidity IgG antibodies as well as DNA, detected by polymerase chain reaction (PCR), were positive in blood, consistent with acute infection. Blood PCRs were negative for adenoviruses and herpes simplex virus. Subsequent functional platelet assays confirmed the presence of platelet‐activating anti‐PF4 antibodies, but heparin and PF4 dependence was variable, depending on the assay; the commercial HITAlert™ assay (+/− additional PF4) showed a classical HIT‐like, heparin‐dependent platelet activation (Figure 1A); SRA showed PF4‐ and heparin‐independent activation that was inhibited by FcɣRIIA blockade with IV.3 IgG and by high but not low concentrations of heparin (Figure 1B); platelet aggregation studies showed classical PF4‐dependent, VITT‐like platelet activation blocked by 0.5 U/mL heparin and IV.3 F(ab)_2_ (Figure 1C,D). Because of these conflicting functional results, epitope mapping and clonality studies for the anti‐PF4 antibodies were performed. Epitope mapping showed antibody binding to four amino acids (H23, N47, K50, K62 [in red in Figure 2A]) previously identified as part of the PF4‐dependent VITT antibody‐binding region, closely aligning with the heparin‐binding site on PF4. Antibodies also recognized three amino acids (A32, C52, L53 [in blue in Figure 2A]) within the PF4‐independent VITT antibody‐binding region, overlapping with the binding sites of the HIT‐like monoclonal antibody KKO. Additionally, antibodies bound to five amino acids outside the VITT epitopes, including two previously undescribed positively charged residues (K61, K55 [in yellow in Figure 2A]) near the heparin‐binding region. Mass spectrometry‐based clonality studies using antibody isolation with both PF4 and PF4/Heparin showed a single IgGλ monoclonal antibody (a single IGHV3‐13*04‐encoded heavy chain paired with a single IGLV4‐60*03‐encoded light chain) with a distinct clonotype to the stereotypic VITT and VITT‐like antibodies that have been previously described (Figure 2B).^14^, ^19^ Despite different molecular signatures, these anti‐PF4 antibodies presented highly similar negatively charged paratopes as VITT antibodies (Figure 2C). Serum protein electrophoresis (SPE) revealed a monoclonal IgGλ band of <1 g/L. Clonotyping of this paraprotein revealed it to be the same IgGλ anti‐PF4 antibody.

## DISCUSSION

This case report details a very unfortunate patient with aggressive thrombosis and thrombocytopenia secondary to monoclonal anti‐PF4 antibodies, with functional testing showing cross‐over VITT‐like and HIT‐like activity. Unique features are the temporal relation to CMV infection, the novel PF4 epitope and the markedly high monoclonal antibody titre (0.2–1 g/L). While high levels of anti‐PF4 paraprotein (4–18 g/L) have been reported in patients with monoclonal gammopathy of thrombotic significance,^20^, ^21^, ^22^, ^23^ all of these patients had chronic presentations, with only mild thrombocytopenia and no preceding viral infections. None of the described VITT‐like antibodies developing post adenoviral infection had detectable paraprotein.^15^, ^16^, ^17^, ^18^ It is possible that the high antibody titre interfered with the functional platelet testing and contributed to the discrepant results.

Thrombosis with thrombocytopenia syndromes (TTS) are often aggressive and potentially fatal. In patients without recent heparin exposure, investigations are typically focussed on identifying pathophysiology, including catastrophic antiphospholipid syndrome, cancer‐associated disseminated intravascular coagulation and PNH. Given recent reports of VITT‐like antibodies identified in TTS samples taken prior to the COVID‐19 pandemic,^8^ descriptions of adenovirus‐induced anti‐PF4‐associated immune thrombocytopenia and thrombosis^13^, ^15^, ^16^, ^17^, ^18^ and now this case with a CMV trigger, it is clear that anti‐PF4 mediated TTS is probably more common than previously thought.

We therefore advocate the British Society of Haematology (BSH) Expert Haematology Panel recommendations to test for anti‐PF4 antibodies in patients with thrombocytopenia, thrombosis and a D‐dimer of >4000 ng/mL regardless of vaccine exposure.^11^ It is important to note that cases of VITT test negative with rapid HIT chemiluminescence tests and therefore must be further tested by ELISA. A chemiluminescence test for VITT is in development, however.^8^ It is also important to emphasize that VITT and HIT are part of a spectrum of anti‐PF4 mediated TTS, as evidenced by this case which has concurrent features of both conditions. Antibody binding to overlap regions between classical VITT and HIT PF4 epitopes may well explain the apparently conflicting results of the different functional platelet activation tests in this patient.

The wide spectrum of different anti‐PF4 mediated TTS is becoming more apparent, and it is not always possible to classify all patients into separate HIT, VITT and various subtype diagnostic categories. We propose a name of PF4‐associated immune thrombocytopenia and thrombosis (PITT) as an overarching term.

We advocate following the treatment algorithms for VITT in all cases of PITT. Even though heparin is effective for adenovirus‐associated VITT‐like syndrome, given our patient's mixed results and clinical deterioration after heparin dose escalation, we suggest initial treatment with non‐heparin anticoagulation if PITT is suspected, before the subtype is identified. Further study is required to delineate optimal treatments for this under‐recognized and life‐threatening condition, such as blockade of FcɣRIIA or its downstream signalling on neutrophils and platelets.^24^

## AUTHOR CONTRIBUTIONS

P. L. R. Nicolson conceived the study, performed experiments and interpreted data, wrote and edited the manuscript. S. J. Montague and R. J. Buka performed experiments, wrote and edited the manuscript. A. Calvert, J. I. Sheppard, Y. Zhang and J. J. Wang performed experiments and data analysis and edited the manuscript. J. Sharman, E. Hassan, J. Harrison, E. Lawrence, P. El‐Dalil and D. Parekh edited the manuscript. H. Osman, J. J. Wang, T. P. Gordon, I. Nazy and T. E. Warkentin supervised experiments, interpreted data and edited the manuscript. W. A. Lester conceived the study, supervised experiments, interpreted data and edited the manuscript.

## FUNDING INFORMATION

S. J. Montague is supported by a British Heart Foundation (BHF) project grant (PG/23/11230, AA/18/2/34218). R. J. Buka is supported through a BHF dedicated scholarship. I. Nazy is supported by grants from the Canadian Institutes of Health Research (363046), The Public Health Agency of Canada, and the Heart and Stroke Foundation of Canada (G‐23‐0035035). JJW is supported by a grant from the Flinders Health and Medical Research Institute.

The National Institute for Health and Care Research (NIHR) Biomedical Research Centre (NIHR203326) has supported the University of Birmingham Department of Cardiovascular Sciences, where this research is based. The opinions expressed in this article are those of the authors and do not represent any of the listed organizations.

## CONFLICT OF INTEREST STATEMENT

PLRN has received research support from Astra Zeneca, Novartis, Rigel and Sanofi, has provided consultancy services for Sobi and has received speaker fees from Astra Zeneca, Bayer, Sobi & Takeda. RJB has received research support from Astra Zeneca. TEW has received research support from Werfen and has provided consulting services for Instrumentation Laboratory Company, Octapharma, Paradigm Pharmaceuticals, Veralox Therapeutics and Wefen. WAL has received speaker fees from Astra Zeneca, Bayer, Bristol‐Myers Squibb, Baichi Sankyo and Pfizer.

## Supporting information


Data S1.
