# Supplementary material for: Anti‐PF4 mediated thrombocytopenia and thrombosis associated with acute cytomegalovirus infection displays both HIT‐like and VITT‐like characteristics
Source: Br J Haematol. 2025 Apr 29;206(6):1737–42. doi: 10.1111/bjh.20092 (PMC12166337; doi:10.1111/bjh.20092)
Supplement: Supplementary file 1 — Data S1. [file BJH-206-1737-s001.docx]

## Supplementary Material

## Supplementary Methods

**Preparation of Platelet Rich Plasma and Washed Platelets**

Blood was taken from consenting healthy, drug-free volunteers, previously shown to be ‘high responders’ to HIT serum ±heparin, into 4% sodium citrate. For preparation of Platelet Rich Plasma (PRP); citrated whole blood was centrifuged at 100 *g* for 5 min at room temperature.

For preparation of washed platelets; citrated (3.2-4%) whole blood was gently mixed with 10% volume acid citrate dextrose (ACD) prior to centrifugation at 200 *g* for 20 min at room temperature. The obtained supernatant PRP was further centrifuged at 1000 *g* for 10 min following addition of 0.2 μg/mL prostacyclin (PGI_2_). Following this, the supernatant was discarded and the platelet pellet resuspended in modified Tyrode’s-HEPES buffer (134 nM NaCl, 0.34 mM Na_2_HPO^4^, 2.9 mM KCl, 12 mM NAHCO_3_, 20 mM HEPES, 5 mM glucose, 1 mM MgCl_2_; pH 7.2). This solution was centrifuged again at 1000 *g* for 10 min following addition of 0.2 μg/mL PGI_2_. The pellet was then resuspended in modified Tyrode’s-HEPES buffer to concentrations outlined below. Platelets were then allowed to rest at room temperature for 30 min prior to use in any experiments.

**HIT ELISA**

Clotted blood was centrifuged at 2000 *g* for 10 min and serum isolated. Anti-PF4 antibodies were measured (in presence or absence of 0.5 U/mL Heparin) using the Immucor LIFECODES™ PF4 IgG anti-PF4/heparin enzymatic immunoassay (EIA, Immucor GTI Diagnostics, Norcross GA).

**HITAlert™️**

The HITAlert™️ kit (IQ Products, Groningen, The Netherlands) was adapted for this assay. Briefly, 10 μL of patient serum was added to 10 μL PRP which was diluted with test buffer in the presence or absence of 10 μg/mL PF4, without or with heparin (0.5 U/mL or 1000 U/mL) to a final volume of 50 μL. These samples were incubated at room temperature on a horizontal orbital shaker for 1 h. A fluorophore (FITC) conjugated platelet activation antibody was added and incubated in the dark for 15 min at room temperature before analysis by flow cytometry.

**Serotonin Release Assay (SRA)**

We performed a washed platelet activation assay, the serotonin-release assay (SRA) as has been previously described,^1^ but also including a modification in which PF4 (10 µg/mL) is added, rather than heparin.^2^ Briefly platelets in PRP was labelled with ^14^C-Serotonin and then washed in Tyrode’s buffer and resuspended to a concentration of 3 x10^8^/mL. 20 μL of heat-treated serum and 5 μL of PF4/heparin were added to wells of a 96-well plate containing 75 μL of ^14^C-Serotonin-loaded washed platelets. Inhibition of reactivity in the presence of Fc receptor-blocking monoclonal antibody (IV.3; 10 μg/mL) was also assessed.

**Platelet Aggregation**

Washed platelets (2 x10^8^/mL) were added to cuvettes and warmed to 37 °C in a Model 490 Aggregometer (ChronoLog, Havertown, PA) for 2 min. For conditions with PF4/heparin or IV.3 F(ab)_2_, platelets were pre-treated for 5 min. Under stirring conditions (1200 rpm) platelets were stimulated with serum (14:1, v/v) for 30 min at 37 °C in the presence of 10 μg/mL PF4 (ChromaTec GmBH, Greifswald, Germany) or vehicle (Phosphate Buffered Saline [PBS]), with or without 0.5 U/mL Heparin (Leo Pharma Inc. Toronto, ON) or 10 μg/mL IV.3 F(ab)_2_ (manufactured in-house). Data presented as area under the curve calculated with Aggrolink 8 software (ChronoLog, Havertown, PA).

**Epitope Mapping**

This technique has been described in detail previously.^3^ Briefly the DNA coding sequence of PF4 was cloned into the pET22b expression vector (GenScript, Piscataway, NJ) and PF4 single amino acid mutants were designed such that non-alanine amino acids were mutated to alanine and alanine amino acids were mutated to valine. PF4 mutants were introduced into E. coli ArcticExpress (DE3) cells (Agilent Technologies, Santa Clara, CA) and overexpressed in cultures before lysis by sonication in 20 mM sodium phosphate, pH 7.2, 400 mM sodium chloride, 1.4 mM β-mercaptoethanol, 5% (v/v) glycerol, 1% (v/v) Triton X-100 (ThermoFisher Scientific, Waltham, MA), and 0.5% (w/v) sodium deoxycholate (Sigma-Aldrich, St. Louis,

MO) with 2 mM MgCl_2_, 10 μg/mL DNaseI (Sigma-Aldrich, St. Louis, MO) and EDTA-free protease inhibitor cocktail (Roche, Basel, Switzerland). The supernatant was cleared by centrifugation (40000 *g*, 40 min) at 4 °C and purified using HiTrap Q HP and then HiTrap Heparin HP columns. 5 μg/mL purified fractions containing wild type or mutant PF4 were applied to create a modified PF4-heparin IgG specific enzyme immunoassay on 384-well NUNC Maxisorp plates (ThermoFisher Scientific, Waltham, MA). In this assay patient serum diluted 1/50 in 1% BSA was added to the wells containing each different wild type and mutant PF4 and incubated for 1 h at room temperature. This was then washed prior to addition of 1:3000 alkaline phosphatase conjugated goat anti-human IgG (Jackson ImmunoResearch Laboratories Inc, Westgrove, PA) for 1 h at room temperature. Then 1 mg/mL p-nitrophenylphosphate (PNPP, Sigma-Aldrich, St. Louis, MO) substrate dissolved in 1 M diethanolamine buffer (pH 9.6) was added to allow detection. Optical density (OD) was measured at 405 nm using a BioTek 800TS microplate reader (BioTek, Winooski, VT) to measure binding of antibodies to PF4.

**Antibody clonality studies**

Anti-PF4 antibodies were purified from patient serum using PF4 protein-coupled MyOne Carboxylic Acid Dynabeads (ThermoFisher)^4^  and anti-PF4/heparin antibodies were isolated using heparin-biotin PF4 complex on streptavidin dynabeads (Invitrogen). The bound antibodies were eluted using 100mM glycine elution buffer (pH 11) and separated by SDS-polyacrylamide gel electrophoresis (PAGE) (criterion stain-free TGX gels; Bio-Rad, Hercules, CA, USA). In-gel tryptic and chymotryptic digests were performed on the IgG gel bands and peptides analysed using an Orbitrap Exploris 480 mass spectrometer (Thermo Scientific) coupled to an Ultimate 3000 UHPLC (Dionex, USA). Peptide sequences were analysed by de novo sequencing and International ImMunoGeneTics (IMGT) database matching using Peaks studio XPro software (Bioinformatics Solution Inc., Waterloo, ON, Canada). Parameters for database searches, data refinement and Ig variable region subfamily assignments were described previously^4–6^. A false discovery rate (FDR) threshold of 1.0% was applied at the peptide level to each data set. High-quality de novo peptides were selected based on sequences having an average local confidence score threshold greater than or equal to 75% and inspected manually to ensure correct assignments. The complementarity-determining region 3 (CDR3) sequences were identified by de novo sequencing derived from different enzyme digested peptides. The Ig variable region subfamily is assigned from the presence of a unique peptide corresponding to the subfamily. Paraprotein was isolated from serum protein electrophoresis gel and sequenced as described above.

## Case Presentation

A 37-year-old caucasian female with no significant medical history presented with five days of general malaise and fevers followed by one-day of abdominal pain. Investigations on admission showed an isolated thrombocytopenia (51 x 10^9^/L) with an otherwise normal full blood count. Blood film confirmed thrombocytopenia and showed neutrophil hypergranulation but no red cell fragmentation. Initial clotting studies showed a slightly raised prothrombin time ratio (1.3), a very high D-dimer (8249 ng/mL) and low fibrinogen (0.66 g/L) consistent with disseminated intravascular coagulation. Biochemical tests showed an isolated raised alanine transaminase (165 u/L) and a mildly raised C-reactive protein (22 mg/L). A full panel of investigation results are shown in supplementary Table 1.

Computed tomography (CT) imaging of the abdomen and pelvis showed pancreatic inflammation, poor contrast opacification of the portal vein and 16 cm splenomegaly. A subsequent doppler ultrasound scan confirmed portal vein thrombosis. Considering the presence of thrombosis, low fibrinogen and low platelet count but absence of bleeding, prophylactic dose low molecular weight heparin (LMWH) was started (enoxaparin 40 mg daily). Tests for underlying causes of unusual site thrombosis including antiphospholipid syndrome, paroxysmal nocturnal haemoglobinuria (PNH) and myeloproliferative disease were performed and subsequently found to be negative. She had no bleeding following prophylactic LMWH and so treatment was escalated to therapeutic LMWH the next day (enoxaparin 140 mg daily). 36 hours after presentation she suddenly became unresponsive and profoundly hypotensive. Further investigations revealed an acute right middle cerebral artery territory infarct. A CT venogram ruled out cerebral venous sinus thrombosis (CVST). A simultaneous CT chest and abdomen demonstrated hepatic and splenic infarction and extensive thrombosis of the entire portal system causing progressive small bowel venous thrombosis and ischaemia necessitating an emergency small bowel resection and a switch to unfractionated heparin (UFH). By this stage she was critically unwell requiring multi-organ support with biochemical evidence of severe liver injury. The aggressive nature of the thrombosis and constellation of laboratory results triggered consideration of PF4-associated immune thrombocytopenia and thrombosis; intravenous methylprednisolone was given and testing for anti-PF4 antibodies was instigated. Chemiluminescence testing was negative, but ELISA testing was highly positive (>3.0 OD) with 98% neutralisation with 0.5 U/mL heparin. These results were identical to those seen with VITT but there was no history of vaccination or heparin exposure within the preceding six months. The patient was then planned for a switch to non-heparin anticoagulation with argatroban and to undergo plasma exchange but before this could happen she suffered a cardiac arrest. Pulmonary embolism was felt to be the likely cause due to a new right bundle branch block and hypoxia. Thrombolysis was contraindicated due to recent stroke and major abdominal surgery and unfortunately the patient was unable to be resuscitated. She died only four days after admission.

Virology results available post-mortem were negative for COVID, Respiratory Syncytial Virus, Influenza A and B, HIV, Hepatitis B and C Virus. Epstein Barr Virus nuclear antigen (EBNA) antibodies were positive indicating past EBV infection. CMV IgM and low avidity IgG antibodies as well as polymerase chain reaction (PCR) (446 iU/mL) were positive in blood, consistent with acute CMV infection. Blood PCRs were negative for adenoviruses and herpes simplex virus. Subsequent functional platelet assays confirmed the presence of platelet-activating anti-PF4 antibodies but heparin- and PF4-dependence was variable and depended on the assay used; commercial HITAlert™ (with and without additional PF4) assays showed a classical HIT-like, heparin-dependent antibody (Figure 1A); serotonin release assay (SRA) showed PF4- and heparin-*in*dependent platelet activation that was blocked by the FcɣRIIA blocking antibody IV.3 and by high but not low concentrations of heparin (Figure 1B); platelet aggregation studies showed a classical VITT-like antibody that activated platelets in combination with PF4, which was blocked by 0.5 U/mL heparin and IV.3 F(ab)_2_ (Figure 1C-D). Because of these conflicting functional results, epitope mapping and clonality studies for the anti-PF4 antibodies were performed. The epitope mapping showed antibodies that bound to four amino acids (H23, N47, K50, K62 [in red in Figure 2A]) previously identified as part of the PF4-dependent VITT antibody-binding region, closely aligning with the heparin-binding site on PF4. Antibodies also recognised three amino acids (A32, C52, L53 [in blue in Figure 2A]) within the PF4-independent VITT antibody-binding region, overlapping with the binding sites of the HIT-like monoclonal antibody KKO. Additionally, antibodies bound to five amino acids outside the VITT epitopes, including two positively charged residues (K61, K55 [in yellow in Figure 2A]) near the heparin-binding region, which have not been described before. Mass spectrometry-based clonality studies using antibody isolation with both PF4 and PF4/Heparin showed a single IgGλ monoclonal antibody (a single IGHV3-13*04-encoded heavy chain paired with a single IGLV4-60*03-encoded light chain) with a distinct clonotype to the stereotypic VITT and VITT-like anti-PF4 antibodies that have been previously described (Figure 2B).^15,20^ Despite different molecular signatures, these anti-PF4 antibodies presented highly similar negatively-charged paratopes as VITT antibodies (Figure 2C). Serum protein electrophoresis (SPE) revealed a monoclonal IgGλ band with a quantification of < 1 g/L. Clonotyping of this paraprotein revealed it to be the same IgGλ anti-PF4 antibody.

## Supplementary Results

|  | **Normal range** | **Day 1** | **Day 2** | **Day 3** | **Day 4** | **Day 5** |
| --- | --- | --- | --- | --- | --- | --- |
| Haemoglobin (g/L) | 120-154 | 124 | 112 | 117 | 103 | 74 |
| WBC (x10^9^/L) | 3-10.9 | 8.2 | 7.6 | 12.1 | 44.3 | 48.7 |
| Platelets (x10^9^/L) | 150-450 | 51 | 48 | 55 | 37 | 44 |
| PT ratio | 0.8-1.2 | 1.3 | 1.4 | 1.3 | 1.7 | 4.7 |
| APTT ratio | 0.8-1.2 | 1 | 1.2 | 1 | 1.1 | 2.4 |
| Fibrinogen (g/L) | 1.5-4.1 |  | 0.66 |  | 1.24 | 0.69 |
| D-dimer (ng/mL) | 0-250 | 8249 |  |  | 3267 |  |
| CRP (mg/L) | 0-5 | 22 |  | 98 | 103 | 118 |
| ALT (U/L) | 5-50 | 165 |  | 114 | 1810 | >6000 |
| Bilirubin (μmol/L) | <21 | 14 |  | 11 | 17 | 32 |
| Ferritin (μg/L) | 20-235 | 200 |  |  | 5839 |  |

**Supplementary Table 1: Blood parameter results during admission:** Abbreviations: Total white blood cell count (WBC). Prothrombin time (PT). Activated Partial Thromboplastin Time (APTT). C-reactive protein (CRP). Alanine Transaminase (ALT). Abnormal results highlighted in Red.

## Supplementary References

1. Sheridan D, Carter C, Kelton JG. A Diagnostic Test for Heparin-Induced Thrombocytopenia. Blood. 1986;67(1):27–30.

2. Nazi I, Arnold DM, Warkentin TE, Smith JW, Staibano P, Kelton JG. Distinguishing between anti–platelet factor 4/heparin antibodies that can and cannot cause heparin‐induced thrombocytopenia. J Thromb Haemost. 2015;13(10):1900–7.

3. Huynh A, Kelton JG, Arnold DM, Daka M, Nazy I. Antibody epitopes in vaccine-induced immune thrombotic thrombocytopaenia. Nature. 2021;596(7873):565–9.

4. Wang JJ, Armour B, Chataway T, Troelnikov A, Colella A, Yacoub O, et al. Vaccine-induced immune thrombotic thrombocytopenia is mediated by a stereotyped clonotypic antibody. Blood. 2022 Oct 13;140(15):1738–42.

5. Wang JJ, Colella AD, Beroukas D, Chataway TK, Gordon TP. Precipitating anti‐dsDNA peptide repertoires in lupus. Clin Exp Immunol. 2018;194(3):273–82.

6. Arentz G, Thurgood LA, Lindop R, Chataway TK, Gordon TP. Secreted human Ro52 autoantibody proteomes express a restricted set of public clonotypes. J Autoimmun. 2012;39(4):466–70.
